# Physical Health Problems as a Suicide Precipitant: Associations With Other Risk Factors and Suicide Methods in Three Age Groups of Older Decedents

**DOI:** 10.1093/geroni/igad073

**Published:** 2023-07-06

**Authors:** Namkee G Choi, Bryan Y Choi, C Nathan Marti

**Affiliations:** Steve Hicks School of Social Work, University of Texas at Austin, Austin, Texas, USA; Department of Emergency Medicine, Philadelphia College of Osteopathic Medicine, Philadelphia, Pennsylvania, USA; Department of Emergency Medicine, BayHealth, Dover, Delware, USA; Steve Hicks School of Social Work, University of Texas at Austin, Austin, Texas, USA

**Keywords:** Depressed mood, Firearms, Physical health problems, Poisoning, Psychiatric disorder

## Abstract

**Background and Objectives:**

Physical health problems are a significant late-life suicide precipitant. This study’s purpose was to examine differences in (i) other suicide precipitants and psychiatric/substance use problems, and (ii) suicide methods (firearms, hanging/suffocation, and poisoning) in 3 age groups (55–64, 65–74, and 75+) of older suicide decedents who had physical health problems as a suicide precipitant.

**Research Design and Methods:**

Data came from the 2017–2019 U.S. National Violent Death Reporting System (*N* = 34,912; 27,761 males [79.5%] and 7,151 females [20.5%]). Generalized linear models for a Poisson distribution with a log link were used to examine the study questions.

**Results:**

Physical health problems were a suicide precipitant for 25.8%, 41.9%, and 57.7% of the 55–64, 65–74, and 75+ age groups, respectively, and were associated with a higher likelihood of having had depressed mood (IRR = 1.38, 95% CI: 1.33–1.43) and other substance use problems (IRR = 1.22, 95% CI: 1.13–1.31). Interaction effects showed that when job/finance/housing problems, depressed mood, or any psychiatric disorders were co-present with physical health problems, the age group differences in the predicted rates of physical health problems were diminished. Physical health problems were also positively associated with firearm and poisoning use, but negatively associated with hanging/suffocation. Interaction effects indicated that the predicted rates of firearm and poisoning use significantly increased among those aged 55–64 with than without physical health problems.

**Discussion and Implications:**

In all 3 age groups of older suicide decedents, physical health problems were the predominant suicide precipitant, and those with physical health problems had elevated depressed mood. Assessment of suicide risk, affordable and accessible health, and mental health services, restriction of access to lethal suicide methods, and policy-based suicide prevention approaches for older adults with physical health problems are needed.

**Translational Significance**: Physical health problems are a significant late-life suicide risk factor, eclipsing other risk factors. Older suicide decedents with than without physical health problems as a suicide precipitant had higher rates of depressed mood and firearm and poisoning use, especially in the 55–64 age group. These findings point to the importance of targeted suicide prevention approaches for those with physical health problems. Access to high-quality physical and mental health care and social services, as well as best clinical practices on limiting access to lethal means and firearm regulations aimed at reducing suicide risk, are needed in a rapidly aging society.

Suicide rates among older adults, especially men 75 years and older, have continued to increase over the past two decades and have been the highest of all age groups (Garnett et al., 2022; Stone et al., 2023). High late-life suicide rates are attributed to a greater intent to die, greater premeditation, and use of more deadly methods, firearms in particular, among older adults compared to younger age groups (Conner et al., 2019; Conwell et al., 1998).

Research on late-life suicidal ideation and behaviors has shown significant risk factors to be male sex; physical health problems; (e.g., terminal/debilitating illnesses and chronic pain); psychiatric disorders; bereavement; living alone; limited social connectedness; reduced social support; psychiatric comorbidity (e.g., depression and anxiety and psychiatric disorders with substance use problems); psychiatric and physical multimorbidity; cognitive impairment; and a diagnosis of Alzheimer’s and related dementia (Ahmedani et al., 2017; Beghi et al., 2021; Conejero et al., 2018; Fässberg et al., 2012, 2016; Fernandez-Rodrigues et al., 2022; Gujral et al., 2021; Harrison et al., 2010; Racine, 2018; Richard-Devantoy et al., 2021; Schmutte et al., 2022; Szanto et al., 2020).

Studies have shown that physical health problems (PH), in particular, terminal illnesses and associated functional limitations and loss of independence, are especially significant late-life suicide precipitants and may explain why older than younger adults have higher suicide rates (Conwell et al., 2002; Erlangsen et al., 2015; Fiske et al, 2008; Harwood et al., 2006; Hawton & Harriss, 2006). Given the increasing rates of chronic/terminal illnesses with advancing age (Buttorff et al., 2017), it is not surprising that the rates/probabilities of PH as a suicide precipitant also increase with age. Studies of middle-aged and older suicide decedents in Queensland, Australia found that relationship problems, financial and legal stressors, psychiatric problems, and suicidal behavior history decreased significantly with age, whereas PH and bereavement increased with age (Koo et al., 2017a, 2017b). Previous studies of suicide decedents in the U.S. National Violent Death Reporting System (NVDRS) also found that PH was a suicide precipitant among a little more than half of those age 65+, with increased predicted probability at advanced ages and among males than females (Choi et al., 2019; Phillips & Hempstead, 2022). Choi et al.’s (2019) examination of coroner/medical examiner and law enforcement reports of suicide decedents age 65+ also showed that bodily pain and cancer, followed by cognitive and functional decline, refusal/fear of nursing home placement, and loss of independence, were the most commonly stated PH suicide precipitants. In addition to being older, suicide decedents with PH as a suicide precipitant, compared to those without, were more likely to have had depressed mood at the time of injury, disclosed their suicidal intent, and used firearms as a suicide method (Choi et al., 2019; Hempstead et al., 2013; Phillips & Hempstead, 2022).

Because suicide is often precipitated by multiple bio-­psycho-social risk factors, PH-related suicides may also result from a combination of other life stressors such as interpersonal conflicts, financial and legal problems, loss and grief, psychiatric disorders, and alcohol and other substance use problems. For example, one study of U.S. adult suicide decedents showed that the rates of job loss, financial stress, and/or eviction as a suicide precipitant were higher in the 45–64 age group than in the younger or older age groups; however, these problems were still a precipitating factor for nearly 10% of decedents age 65+ (Choi et al., 2022). More research is needed to better understand how other risk factors may be associated with PH in different age groups of U.S. older-adult suicide decedents. Identifying key suicide precipitants among different age groups of older adults is important for developing and applying better-targeted suicide prevention approaches. To that end, we also need to examine whether or not PH may be associated with different suicide methods in different age groups.

In the present study, based on the 2017–2019 NVDRS data, our research questions were whether or not three age groups (55–64, 65–74, and 75+) of older suicide decedents who had PH as a suicide precipitant may differ on (i) other suicide precipitants and psychiatric/substance use problems; and (ii) suicide methods (firearms, hanging/suffocation, and poisoning). Based on previous study findings on different types of suicide precipitants in different age groups (Choi et al., 2019, 2022; Koo et al., 2017a, 2017b), the study hypotheses were that PH in the 65–74 and 75+ age groups than in the 55–64 age group would be associated with: (*H1*) a lower likelihood of relationship problems, job/finance/housing problems, psychiatric disorder, and alcohol and substance use problems; and (*H2*) a higher likelihood of firearm use but a lower likelihood of hanging/suffocation and poisoning. The study findings will provide more insight into older decedents whose suicide was in part precipitated by PH and empirical data needed for developing effective late-life suicide prevention approaches.

## Method

### Data Source

The NVDRS is the only state-based violent death reporting system in the U.S. that provides information and context on when, where, and how violent deaths occur and who is affected (National Center for Injury Prevention and Control [NCIPC]; NCIPC, 2021). NVDRS links data from death certificates and reports from coroners/medical examiners and law enforcement agencies on cases of violent deaths: suicides, homicides, deaths from legal intervention (i.e., victims killed by law enforcement acting in the line of duty), deaths of undetermined intent, and unintentional firearm deaths. Coroner/medical examiner and law enforcement reports are from the injury/death scene, ongoing investigations, or family/friend accounts, and often serve as the basis of the circumstances of death as well as NVDRS variables that were “calculated” (coded “Yes” when endorsed by the coroner/medical examiner and/or law enforcement reports vs “No/not available/unknown”). These “calculated” variables include one or more suicide precipitants, depressed mood, mental health diagnoses, substance use problems, prior suicidal thoughts and behaviors, recent suicidal intent disclosure, and mental health or substance use treatment history. When available, crime lab and toxicology reports included in coroner/medical examiner reports are also abstracted and entered in NVDRS.

We used 2017–2019 NVDRS data because the number of participating states increased from 27 in 2016 to 37 in 2017 and to 43 states, the District of Columbia, and Puerto Rico in 2019, although not all states provided complete data for all three years (NCIPC, 2020). Our preliminary analysis showed that some important results vary depending on the number of participating states. The authors of this study were granted access to de-identified NVDRS data, 2017–2019, by the Centers for Disease Control and Prevention’s NVDRS-Restricted Access Data review committee. This study based on de-identified decedents (*N* = 34,912 age 55+; 27,761 males [79.5%] and 7,151 females [20.5%] participants) was exempt from the authors’ institutional review board’s review.

### Measures

#### Demographic variables

Data on age at the time of death, sex, race/ethnicity, marital status, level of education, and Veteran status were from the death certificates and coroner/medical examiner reports. In this study, decedents were categorized into 55–64, 65–74, and 75+ age groups to examine age group differences, and the 55–64 age group was used as the reference category in multivariable models because this age group was less likely to have had PH as a suicide precipitant than the 65–75 and 75+ age groups. We also included the U.S. census region (Northeast, Midwest, South, West, Puerto Rico, and U.S. territories) of decedents’ residence to control for possible geographic differences in suicide methods.

#### PH as a suicide precipitant

PH was coded as a suicide precipitant only if any diagnosed or perceived physical health problem (e.g., terminal disease, chronic pain, and debilitating condition) was relevant to the death (e.g., “despondent over recent diagnosis of cancer” or “complained that he could not live with the pain associated with a condition” even if the condition may not have been diagnosed or existed).

#### Other suicide precipitants

These included: (i) *relationship problems* (conflict with an intimate partner or conflict with other family members, arguments, other family stressors, caregiver burden, or abuse by a caregiver); (ii) *job/finance/housing problems*: Job problems refer to job loss (fired or laid off) and/or difficulties finding/keeping a job or difficulties with demotion or serious conflict/stress at work. Financial problems refer to financial difficulties, e.g., bankruptcy, overwhelming debts, or foreclosure of a home or business. Eviction or loss of housing refers to a recent eviction or other loss of the victim’s housing, or the threat of it. Although the NVDRS coded job problems, financial problems, and eviction or loss of housing separately, we combined them into a single category in this study given that these problems tend to occur in step with one another (i.e., job loss leading to financial problems and finally, and loss of housing); (iii) *criminal/civil legal problems*: Criminal legal problems refer to conducts (including driving under the influence) with negative legal or law enforcement consequences (e.g., about to enter jail, facing a court date, on the run from law enforcement). Civil legal problems refer to a divorce, custody dispute, civil lawsuit, or legal problems, and suicide/death of family/friend in NVDRS. Criminal legal problems refer to conduct (including driving under the influence) with negative legal or law enforcement consequences (e.g., about to enter jail, facing a court date, on the run from law enforcement); and (iv) *suicides or other deaths of family/friends* were coded “Yes” if the decedent was distraught over or reacting to a suicide or other death of a friend or family member or to an anniversary of the suicide or other death.

#### Psychiatric and substance use problems

In NVDRS, psychiatric and substance use problems were coded “Yes” without the need for any indication that they directly contributed to the death and included the following: (i) *depressed mood at the time of injury* (“sad, despondent, down, blue, low, and/or unhappy” mood without the need to have a clinical diagnosis); (ii) *diagnosed psychiatric disorders* and syndromes listed in *DSM-5* (American Psychiatric Association, 2013) at the time of death: shown as the number of diagnoses in descriptive analysis, but as any (=1) versus none (=0) in multivariable models; (iii) *alcohol problem/addiction*; (iv) *other substance use/addiction* (e.g., prescription drug misuse, chronic/abusive/problematic marijuana use, any use of other illicit drugs or inhalants); and (v) *history of suicide attempt* (any previous suicide attempt before the fatal incident, regardless of the severity and injury status).

#### Number of crises

This refers to the number of current/acute events within two weeks of death that the decedent faced with respect to physical health, relationship, and other life stressors as well as psychiatric and substance misuse/addiction problems discussed earlier.

#### Suicide methods

Suicide methods in NVDRS were identified from the International Classification of Diseases, 10th Revision (ICD-10), codes for intentional self-harm (X60−X84) for an underlying cause of death in death certificates and/or from the underlying cause descriptions in CME reports. They included the following: firearms; hanging/suffocation; poisoning due to any type of alcohol/drug/medicine/chemical overdose or with gas (e.g., carbon monoxide and nitrogen); laceration/sharp instruments; blunt objects; jumping from heights; contact with moving objects (train/other vehicles); drowning; and other (fire, hypothermia, electrocution, starvation, dehydration, not adhering to or refusing medical care, or undetermined causes). In this study, we focused on three leading methods: firearms, hanging/suffocation, and poisoning.

We included *injury location, suicidal intent disclosure*, and *mental health or substance use treatment within the preceding 2 months*, for descriptive purposes only. Intent disclosure refers to: (a) disclosure of suicidal thoughts or intent to die by suicide to another person via verbal, written, or electronic communications within a month before suicide (i.e., not at the moment of the suicide), whether explicitly (e.g., “I plan to go to my cabin with my gun and never come back”) or indirectly (e.g., “I know how to put a permanent end to this pain”), or (b) a separate suicide attempt within a month of the suicide. Mental health or substance use treatment included prescription medications, therapy sessions, and/or self-help group participation within 2 months of the injury.

### Analysis

All statistical analyses were performed using Stata/MP 18. First, we used cross-tabulations with Pearson’s χ^2^ tests to describe the three age groups with respect to their other demographic characteristics and the rates of PH, other suicide precipitants, and psychiatric/substance use problems. Second, we used χ^2^ tests to compare those with and without PH as a suicide precipitant in each age group with respect to other suicide precipitants and psychiatric/substance use problems. Third, to test *H1* (associations of PH with other suicide precipitants and psychiatric/substance use problems), we fit five generalized linear models for a Poisson distribution with a log link for: (1) all decedents age 55+; (2) decedents in the 55–64, 65–74, and the 75+ age groups separately; and (3) all decedents age 55+ with interaction effects between age group and other suicide precipitants and psychiatric/substance use problems, controlling for other demographic characteristics and the number of crises. Fourth, to test *H2* (associations of PH with three leading suicide methods), we fit two generalized linear models for a Poisson distribution with a log link for main effects and interactions effects (PH × age group) for each suicide method, controlling for other suicide precipitants, psychiatric/substance use problems, other demographic characteristics, and the number of crises. We conducted posthoc tests for interaction effects using marginal means and plots to facilitate the interpretation of the interaction terms. We used the Poisson distribution with a log link rather than logistic regression models because odds ratios exaggerate the true relative risk to some degree when the event (i.e., PH and suicide methods in this study) is a common (i.e., >10%) occurrence (Grimes & Schulz, 2008). As a preliminary diagnostic, we used the variance inflation factor, using a cutoff of 2.50 (Allison, 2012), from linear regression models to assess multicollinearity among covariates. Variance inflation factor diagnostics indicated that multicollinearity was not a concern. Generalized linear modeling results are reported as incidence rate ratios (IRRs) with 95% confidence intervals (CIs). Significance was set at *p* < .05.

## Results

### Characteristics of Three Age Groups of Suicide Decedents

Table 1 shows that compared to the 55–64 age group, the 65–75 and 75+ age groups (the oldest age being 105 years) included higher proportions of males, non-Hispanic Whites, married individuals, and veterans. Compared to the 55–64 and 65–74 age groups, the 75+ age group included a higher proportion of those without a high school degree. Most injuries, especially among those aged 75+, occurred at home.

**Table 1.** Demographic and Clinical Characteristics of Suicide Decedents by Age Group

Characteristic	55–64 age group	65–74 age group	75+ age group	*p* Value
*N* (%)	16,995 (48.7)	9,797 (28.1)	8,120 (23.2)	
Sex (%)				<.001
Male	75.9	79.3	87.3	
Female	24.1	20.7	12.7	
Race/ethnicity (%)				<.001
Non-Hispanic White	88.8	90.5	92.0	
Black/African American	3.6	2.9	2.1	
Hispanic	4.5	3.8	3.0	
Asian/Pacific Islander	2.0	1.9	2.3	
Other	1.2	0.9	0.6	
Education (%)				<.001
≤High school	51.0	47.8	56.9	
Some college/associate’s degree	23.0	23.0	16.5	
Bachelor’s degree or higher	22.0	26.0	23.9	
Unknown	3.0	3.2	2.7	
Marital status (%)				<.001
Married	38.9	44.3	43.3	
Divorced/separated	36.4	30.2	18.3	
Widowed	55	12.2	32.7	
Never married	17.6	11.7	4.8	
Missing	1.6	1.6	0.9	
Military service history (Veteran; %)	13.7	31.5	50.4	<.001
Region of residence				<.001
Northeast	18.7	16.4	16.7	
Midwest	28.2	25.7	25.8	
South	26.6	27.9	26.3	
West	25.4	28.6	30.1	
Puerto Rico & territories	1.1	1.4	1.1	
Injured at home	72.6	78.7	84.9	<.001
Suicide precipitating factors^a^ (%)				
Physical health problem^b^	25.8	41.9	57.7	<.001
Physical health problem was a crisis	5.2	10.3	15.6	<.001
Intimate partner relationship problem	16.8	10.7	5.5	<.001
Other relationship problem^c^	7.7	6.9	4.3	<.001
Job, finance, and/or housing problem	20.3	11.6	6.6	<.001
Criminal or civil legal problem	8.4	4.7	1.6	<.001
Suicide/death of family/friend or traumatic anniversary	9.1	8.9	12.4	<.001
Number of crises^d^, *M* (*SD*)	0.31 (0.63)	0.28 (0.56)	0.27 (0.51)	<.001
Depressed mood at the time of injury (%)	32.3	31.7	33.0	.191
Number of any recent psychiatric disorder^e^ (%)				<.001
None	53.4	58.4	70.4	
1	30.9	28.4	22.7	
2+	15.7	13.2	6.9	
Alcohol problem/addiction (%)	19.8	12.0	3.9	<.001
Other substance use problem^f^ (%)	11.2	5.0	1.2	<.001
Suicide attempt history (%)	16.9	12.6	7.2	<.001
Disclosed suicidal intent within last month (%)	18.8	18.7	21.4	<.001
Mental health or substance use treatment within 2 months	25.7	22.7	14.0	<.001
Suicide method				<.001
Firearms	50.0	62.7	74.6	
Hanging/suffocation	23.1	14.6	9.6	
Poisoning	18.1	15.7	10.4	
Other	8.8	7.1	5.4	

*Notes*: *p* Values are calculated based on omnibus Pearson’s χ^2^ tests for all categorical variables and Analysis of variance (ANOVA) for the number of crises (*F* = 17.84, *df* = 2).

^a^Decedents may have multiple precipitating factors.

^b^Including any terminal/other illness, debilitating condition, chronic/acute pain, or other physical/functional issue (perceived or diagnosed) that were relevant to suicide.

^c^Problems with other family/relatives, other family stressors, caregiver burden, arguments, or abuse by a caregiver.

^d^Total number of precipitating factors and psychiatric/substance use problems that posed a crisis within 2 weeks of death.

^e^Including those disorders and syndromes listed in the Diagnostic and Statistical Manual of Mental Disorders, Fifth Edition (*DSM-5*) with the exception of alcohol and other substance dependence; depressive disorder/dysthymia, bipolar disorder, anxiety disorder, post-traumatic stress disorder, and schizophrenia.

^f^Including prescription drug misuse and illicit drug use, even if addiction or abuse is not specifically mentioned. The exception to this is marijuana use. For marijuana, the use must be noted as chronic, abusive, or problematic.

PH was a suicide precipitant for 25.8%, 41.9%, and 57.7% of the 55–64, 65–74, and 75+ age groups, respectively, and was a crisis for 5.2%, 10.3%, and 15.6% of the 55–64, 65–74, and 75+ age groups, respectively. Unlike PH, the rates of relationship, job/finance/housing, and legal problems were lower in the two older groups, but, as expected, the rate of suicide/death of family/friends was the highest in the 75+ age group. Of note, even in the 55–64 age group, PH was more prevalent than these other precipitants.

The three age groups did not differ in the rates of depressed mood at the time of injury; however, two older groups, compared to the 55–64 age group, had lower rates of diagnosed psychiatric disorders, alcohol use problems, other substance use problems, and suicide attempt history. The 75+ age group, compared to the 55–64 and 65–74 age groups, had a higher rate (21.4%) of suicidal intent disclosure within a month before their death. All three age groups had low rates of mental health or substance use treatment receipt within 2 months, with the lowest rate (14.0%) in the 75+ age group.

Firearms were used in 50.0%, 62.7%, and 74.6% of the 55–64, 65–74, and 75+ age groups, respectively; hanging/suffocation was used in 23.1%, 14.6%, and 9.6% of the 55–64, 65–74, and 75+ age groups, respectively; and poisoning was used in 18.1%, 15.7%, and 10.4% of the 55–64, 65–74, and 75+ age groups, respectively.

### Comparison of Those With and Without PH

Table 2 shows that in all three age groups, those with than without PH had lower rates of intimate partner relationship and legal problems. Those with PH in two older age groups also had lower rates of other relationship problems, job/finance/housing problems, suicide/death of family/friends, and prior suicide attempt. In the 55–64 age group, those with PH had higher rates of other substance use problems and psychiatric disorders, but in the two older age groups, those with PH had lower rates of psychiatric disorders. In all three age groups, those with PH had significantly higher rates of depressed mood (e.g., 38.9% vs 25.0% in the 75+ age group) and suicidal intent disclosure (e.g., 25.8% vs 15.3% in the 75+ age group). However, those with PH in all three groups had even lower rates of mental health or substance use treatment receipt (e.g., 13.4% vs 14.9% in the 75+ age group). In all three age groups, those with PH had significantly higher rates of firearm use (e.g., 78.8% vs 68.9% in the 75+ age group).

**Table 2.** Those With and Without Physical Health Problems (PH) as a Suicide Precipitant: Comparison of Other Clinical Characteristics and Suicide Methods

Variable	55–64 age group *n* = 16,995			65–74 age group *n* = 9,797			75+ age group *n* = 8,120		
	No PH 74.2%	Yes PH 25.8%	*p* Value	No PH 58.1%	Yes PH 41.9%	*p* Value	No PH 42.3%	Yes PH 57.7%	*p* Value
Other suicide precipitating factors (%)									
Intimate partner relationship problem	18.6	11.4	<.001	13.8	6.4	<.001	8.2	3.5	<.001
Other relationship problem	7.6	7.9	.558	7.1	6.1	.047	4.6	4.0	.160
Job, finance, and/or housing problem	20.1	20.1	.676	12.4	9.3	<.001	7.3	5.1	<.001
Criminal or civil legal problem	9.5	5.1	<.001	6.5	2.2	<.001	3.0	0.7	<.001
Suicide/death of family/friend or traumatic anniversary	8.9	9.7	.119	9.7	7.9	.002	14.6	10.8	<.001
Psychiatric and substance use problems (%)									
Depressed mood	29.4	40.8	<.001	27.2	38.0	<.001	25.0	38.9	<.001
Any psychiatric disorder	45.8	48.9	.001	42.7	40.0	.008	30.8	28.8	.046
Alcohol problem	20.0	40.8	.160	13.2	10.3	<.001	4.2	3.7	.211
Other substance use problem	10.4	13.5	<.001	4.8	5.3	.204	1.2	1.3	.791
Suicide attempt history	17.0	16.6	.585	13.8	10.9	<.001	8.9	5.9	<.001
Mental health/substance use treatment receipt	25.3	27.0	.031	23.8	21.1	.001	14.9	13.4	.047
Suicidal intent disclosure	17.5	22.5	<.001	15.8	22.6	<.001	15.3	25.8	<.001
Suicide method (%)									
Firearms	47.6	56.9	<.001	57.3	70.1	<.001	68.9	78.8	<.001
Hanging/suffocation	25.5	16.3	<.001	18.0	10.0	<.001	12.0	7.9	<.001
Poisoning	17.2	20.5	<.001	16.1	15.0	.117	11.6	9.6	.004

Additional analyses showed that among those with PH, those with than without depressed mood had significantly higher rates of all other suicide precipitants, alcohol (but not substance use) problems, prior suicide attempts, suicidal intent disclosure, and mental health or substance use treatment receipt. However, those with than without depressed mood were not more or less likely to use firearms.

### Associations of PH With Other Suicide Precipitants and Psychiatric/Substance Use Problems

**Figure 1.**
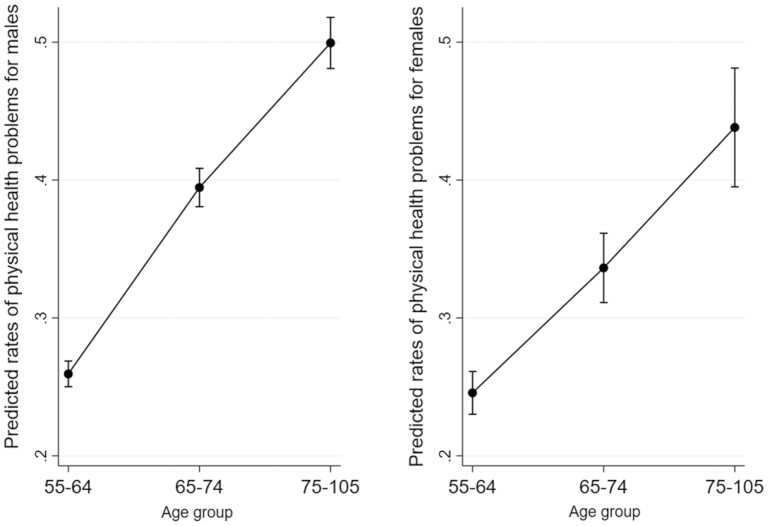
Adjusted predicted rates of physical health problems as a suicide precipitant by age group and sex.

The main effect column of Table 3 shows that the predicted rates of PH as a suicide precipitant were significantly higher in the 65–74 (IRR = 1.49, 95% CI: 1.42–1.55) and 75+ (IRR = 1.89, 95% CI: 1.80–1.99) age groups than in the 55–64 age group. PH was also associated with a higher likelihood of having had depressed mood (IRR = 1.38, 95% CI: 1.33–1.43) and other substance use problems (IRR = 1.22, 95% CI: 1.13–1.31). However, PH was associated with a lower likelihood of having had all other suicide precipitants, alcohol problems, and suicide attempt history. Of the control variables (results not shown in Table 3), the predicted rates of PH were significantly higher among the residents of the Midwest, South, and West, compared to those in the Northeast, and among those with a higher number of crises; however, the rates were significantly lower among females, Blacks, Hispanics, or Asian Pacific Islanders, and nonmarried individuals. Figure 1 shows adjusted predicted rates of PH as a suicide precipitant by sex in three age groups.

**Table 3.** Associations of Physical Health Problems (vs No Physical Health Problems) as a Suicide Precipitant with Other Suicide Precipitating and Contributing Factors: Generalized Linear Modeling Results for Main and Interaction Effects

Variable	Main effect model	Age group separate models			Interaction effect model^a^
	All age 55+ IRR (95% CI)	55–64 age group IRR (95% CI)	65–74 age group IRR (95% CI)	75+ age group IRR (95% CI)	All age 55+ IRR (95% CI)
Age group: vs 55–64 age group					
65–74 age group	1.49 (1.42–1.55)***				1.72 (1.61–1.85)***
75+ age group	1.89 (1.80–1.99)***				2.16 (2.02–2.32)***
Other suicide precipitating factors					
Intimate partner relationship problem vs none	0.52 (0.48–0.56)***	0.53 (0.48–0.58)***	0.49 (0.43–0.56)***	0.55 (0.47–0.64)***	0.52 (0.47–0.58)***
Other relationship problem vs none	0.86 (0.80–0.93)***	0.86 (0.77–0.96)**	0.84 (0.74–0.96)*	0.89 (0.77–1.03)	0.87 (0.78–0.97)*
Job, finance, and/or housing problem	0.79 (0.75–0.84)***	0.85 (0.78–0.92)***	0.72 (0.65–0.80)***	0.74 (0.65–0.85)***	0.83 (0.77–0.90)***
Criminal or civil legal problem	0.46 (0.41–0.51)***	0.51 (0.44–0.59)***	0.42 (0.34–0.51)***	0.35 (0.25–0.50)***	0.50 (0.44–0.58)***
Suicide/death of family/friend or traumatic anniversary	0.80 (0.75–0.86)***	0.88 (0.79–0.98)*	0.78 (0.69–0.88)***	0.77 (0.69–0.85)***	0.88 (0.79–0.98)*
Depressed mood vs none	1.38 (1.33–1.43)***	1.49 (1.39–1.58)***	1.36 (1.27–1.45)***	1.31 (1.23–1.39)***	1.48 (1.39–1.57)***
Any psychiatric disorder vs none	0.98 (0.94–1.02)	1.06 (0.99–1.12)	0.93 (0.87–1.00)*	0.95 (0.91–1.02)	1.05 (0.99–1.12)
Alcohol problem vs none	0.91 (0.85–0.96)**	0.90 (0.83–0.97)**	0.87 (0.78–0.96)*	0.98 (0.84–1.15)	0.90 (0.83–0.97)**
Other substance use problem vs none	1.22 (1.13–1.31)***	1.25 (1.14–1.37)***	1.16 (1.01–1.34)*	1.03 (0.79–1.33)	1.26 (1.15–1.38)***
Suicide attempt history vs none	0.92 (0.87–0.97)**	0.94 (0.87–1.03)	0.90 (0.81–1.00)*	0.86 (0.76–0.98)*	0.95 (0.87–1.03)
Interaction effects					
Job, finance, and/or housing problem × age group					
Job, finance, and/or housing problem × 65–74 age group					0.87 (0.76–0.99)*
Job, finance, and/or housing problem × 75+ age group					0.90 (0.77–1.04)
Depressed mood × age group					
Depressed mood × 65–74 age group					0.92 (0.84–1.01)
Depressed mood × 75+ age group					0.88 (0.81–0.96)**
Psychiatric disorder × age group					
Psychiatric disorder × 65–74 age group					0.89 (0.81–0.97)**
Psychiatric disorder × 75+ age group					0.91 (0.83–0.99)*
*N*	34,912	16,995	9,797	8,120	34,912

*Notes*: All models were adjusted for the following variables: Sex, race/ethnicity, education, marital status, military service status, census region, and the number of crises.

^a^Nonsignificant interaction terms were excluded from the last column: Intimate partner relationship problem × age group; other relationship problem × age group; criminal/civil legal problem × age group; suicide/death of family/friend or traumatic anniversary × age group; alcohol problem × age; other substance use problem × age; and suicide attempt history × age group.

*p < .05; **p < .01; ***p < .001.

The results from separate age-group analyses in Table 3 show that although PH was associated with a lower likelihood of having had an intimate partner relationship, job/finance/housing, legal, and suicide/death of family/friends in all three age groups, other relationship problems were not a significant factor in the 75+ age group. Depressed mood was significantly positively associated with PH in all three age groups; however, having had any psychiatric disorder was negatively associated with PH in the 65–74 age group only. Alcohol problems were significantly negatively associated with, and other substance use problems were significantly positively associated with PH in the 55–64 and 65+ age groups but not in the 75+ age group. Suicide attempt history was significantly negatively associated with PH in the 65–74 and 75+ age groups only.

### Interaction Effects Between Age Group and PH

The last column of Table 3 shows significant interaction effects that were found in job/finance/housing problems, depressed mood, and any psychiatric disorder. The differences in the predicted rates of PH as a suicide precipitant decreased (i) between the 55–64 age group and the 65–74 age group among those with than without job/finance/housing problems (IRR = 0.87, 95% CI: 0.76–0.99); (ii) between the 55 and 64 age group and the 75+ age group among those who had depressed mood (IRR = 0.88, 95% CI: 0.81–0.96) compared to those who did not have depressed mood; (iii) between the 55 and 64 age group and both 65–74 and 75+ age groups among those with than without any psychiatric disorder diagnosis (IRR = 0.89, 95% CI: 0.81–0.97 for the 65–74 age group and IRR = 0.91, 95% CI: 0.83–0.99 for the 75+ age group).

### Associations of PH With Suicide Methods

The main effect model in Table 4 shows that the predicted rates of firearm use were significantly higher among those with than without PH as a suicide precipitant (IRR = 1.13, 95% CI: 1.09–1.16) and in the 65–74 (IRR = 1.13, 95% CI: 1.09–1.17) and 75+ (IRR = 1.18, 95% CI: 1.14–1.23) age groups than in the 55–64 age group. Interaction effects further showed that the differences in the predicted rates of firearm use between the 55and 64 and 75+ age groups decreased when PH was present, as the firearm use rate significantly increased among those aged 55–65 with than without PH (i.e., differences in predicted probability = .08, 95% CI: 0.05–0.10).

**Table 4.** Associations of Suicide Method with Physical Health Problems (PH) as a Suicide Precipitant: Generalized Linear Modeling Results

Variable	Firearms vs no firearms		Hanging/suffocation vs no hanging/suffocation		Poisoning vs no poisoning	
	Main effect IRR (95% CI)	Interaction effect IRR (95% CI)	Main effect IRR (95% CI)	Interaction effect IRR (95% CI)	Main effect IRR (95% CI)	Interaction effect IRR (95% CI)
PH as a suicide precipitant: vs no PH	1.13 (1.09–1.16)***	1.17 (1.11–1.22)***	0.67 (0.63–0.71)***	0.68 (9.63–0.74)***	1.12 (1.05–1.18)***	1.21 (1.12–1.31)***
Age group: vs 55–64 age group						
65–74 age group	1.13 (1.09–1.17)***	1.13 (1.08–1.18)***	0.74 (0.70–0.79)***	0.76 (0.71–0.82)***	0.98 (0.92–1.05)	1.03 (0.95–1.12)
75+ age group	1.18 (1.14–1.23)***	1.23 (1.17–1.30)***	0.58 (0.53–0.64)***	0.57 (0.51–0.63)***	0.81 (0.74–0.88)***	0.89 (0.79–0.99)*
Interaction effects						
PH × 65–74 age group		0.98 (0.92–1.05)		0.90 (0.78–1.04)		0.87 (0.76–0.99)*
PH × 75+ age group		0.92 (0.86–0.98)*		1.06 (0.90–1.24)		0.81 (0.69–0.95)**
*N*	34,912	34,912	34,912	34,912	34,912	34,912

*Notes*: All models were adjusted for the following variables: Sex, race/ethnicity, education, marital status, military service, census region, other suicide precipitating factors, psychiatric disorder, substance use problems, and the number of crises.

*p < .05; **p < .01; ***p < .001.

The main effect model in Table 4 shows that the predicted rates of hanging/suffocation were significantly lower among those with than without PH as a suicide precipitant (IRR = 1.12, 95% CI: 1.05–1.18) as well as in the 65–74 (IRR = 0.74, 95% CI: 0.70–0.79) and 75+ (IRR = 0.58, 95% CI: 0.53–0.64) age groups than in the 55–64 age group. The interaction terms between the age group and PH were not significant.

The main effect model in Table 4 also shows that the predicted rates of poisoning were significantly higher among those with than without PH as a suicide precipitant (IRR = 1.12, 95% CI: 1.05–1.18) but lower among the 75+ age group (IRR = 0.81, 95% CI: 0.74–0.88) than in the 55–64 age group. Interaction terms between age group and PH further showed that when PH was present, the differences in the predicted rates of poisoning use between the 55 and 64 and both 65–74 and 75+ age groups decreased, as poisoning use significantly increased among those age 55–65 with than without PH (i.e., differences in predicted probability = .02, 95% CI: 0.01–0.03).

Of the control variables (results not shown in Table 4), the predicted rates of firearm and hanging/suffocation uses were lower among females than males but the predicted rates of poisoning were 3.3 times higher among females than males. The predicted rates of firearm use were lower among Blacks, Hispanics, or Asian Pacific Islanders and those with a college education but higher among residents of the Midwest, South, and West, compared to those in the Northeast. The predicted rates of hanging/suffocation were higher among Blacks, Hispanics, or Asian Pacific Islanders but lower among those who faced with more crises. Hanging/suffocation and poisoning were also less likely among the residents of the Midwest, South, and West.

## Discussion and Implications

High suicide rates among older adults are a serious public health crisis. In this study of suicide decedents age 55+, we examined the rates of PH as a suicide precipitant and the associations of PH with other suicide precipitants and psychiatric/substance use problems and suicide methods in three age groups. As expected, the proportions that had PH as a suicide precipitant increased with age and was the highest (57.7%) in the 75+ age group, followed by the 65–74 age group (41.9%) and the 55–64 age group (25.8%). Based on previous studies (Choi et al., 2019; Phillips & Hempstead, 2022), the high rates of PH as a suicide precipitant in the 65–74 and 75+ groups were expected. However, the finding that PH was the most prevalent suicide precipitant even in the 55–64 age group points to the importance of paying increased attention to suicide risk among people with PH regardless of age. Research has shown that among young and middle-aged people, followed by those 60+ years, disruptions to daily activity or activity limitations resulting from physical illnesses rather than the number of chronic health conditions predicted suicide risk (Onyeka et al., 2020).

Multivariable analyses confirmed that the predicted rates of PH as a suicide precipitant increased with age, and that those with PH were less likely to have had all other suicide precipitants and alcohol use problems but more likely to have had depressed mood at the time of injury and other substance use problems. The higher likelihood of other substance use problems among those with PH likely reflected the high prevalence of prescription medication (e.g., opioids for pain) use for their PH. Age group separate analyses showed that in all three age groups, PH was associated with a lower likelihood of having had intimate partner relationships, job/finance/housing, legal, and suicide/death of family/friends problems but a higher likelihood of having had depressed mood.

Interaction effects further showed that when job/finance/housing problems, depressed mood, or any psychiatric disorders were co-present with PH, however, age group differences between the 55 and 64 age group and one or both of two older groups in the predicted rates of PH were diminished. This suggests that among some decedents aged 55–64, job/finance/housing problems may have been related to their ill health and disability and that mental health problems (which were more prevalent in this age group) may also have amplified their PH-related distress to a greater extent. PH was positively associated with firearm and poisoning use, but negatively associated with hanging/suffocation. Interaction effects indicated that the predicted rates of firearm and poisoning uses significantly increased among those aged 55–64 with than without PH. These findings partially support *H1* and *H2*.

The study’s key finding is that in all three age groups of older suicide decedents, PH was the predominant suicide precipitant. It is possible that PH became a more predominant suicide precipitant than other precipitants in these older decedents as older adults in general and especially those with serious health problems may have been disengaged from some life areas and associated stressors (e.g., job problems, criminal/legal problems, and alcohol use problems) (Choi et al., 2022; Koo et al., 2017a, 2017b).

Another key finding is the elevated depressed mood close to injury among those with PH. Poor health and functional impairments are significant late-life depression risk factors (de la Torre-Luque et al., 2019; Vyas & Okereke, 2020). Depression, in turn, generates cognitive and interpersonal vulnerabilities (Liu & Alloy, 2010). Thus, depressed mood likely amplified distress related to PH and other life problems, leading to an increased risk of suicide as depressed older adults tend to experience more severe and distressing symptoms from physical ill health and disability, social isolation/loneliness, and feelings of hopelessness (Bickford et al., 2021; Fernandez-Rodrigues et al., 2022).

Despite their elevated depressed mood, only a small proportion of those with PH received any mental health treatment within the preceding 2 months of their death. Our finding is consistent with a recent systematic review that showed low rates of suicide risk help-seeking and service utilization among middle-aged and older adults (Wang et al., 2023). Treatment nonreceipt may have been in part due to unaffordability and other barriers to accessing care (e.g., transportation, provider unavailability, stigma, mistrust of treatment providers; NCIPC, 2021; Wang et al., 2023). Of those who were in treatment, non-adherence to treatment regimen or premature termination may have been a problem (NCIPC, 2021). The fact that a quarter of suicide decedent age 75+ with PH disclosed their suicidal intent within a month of their death underscores the overall inadequacy/lack of effective suicide prevention approaches even for those who may have tried to seek help by disclosing their suicide intent.

Based on previous study findings (Choi et al., 2022; Goldman-Mellor et al., 2021; Hempstead et al., 2013), the associations between PH and firearm use as a suicide method among older adults, especially males, were expected. Our study adds to the knowledge base that PH increases the predicted rate of firearm use among the 55–64 age group, suggesting that intention to die may be stronger among these younger individuals with than without PH. Older adults with than without PH also had a higher rate of poisoning use, and those aged 55–64 with than without PH had increased predicted rates of poisoning use. These younger age individuals may have had easier access to poisons including prescription medications for their medical conditions or other substances. In sum, the influence of PH on choices of suicide methods (i.e., firearm and poisoning uses) among the 55–64 age group should be noted.

The study has a few limitations due to NVDRS data constraints, including the uncertain validity of suicide precipitating factors in NVDRS and the limited generalizability of the findings to NVDRS nonparticipating states. PH and other suicide precipitating factors in NVDRS were based on reports from decedents’ family/friends and other informants and/or suicide notes and could not be independently verified. Although 43 states, the District of Columbia, and Puerto Rico participated in the 2017–2019 NVDRS, some states did not provide data on all three observation years and others provided only partial data limited to some counties. Furthermore, we suspect that the actual number of poisoning suicides was likely higher as suicides by drug overdose could have been misclassified as “accidental” drug overdose deaths as drug overdose deaths escalated over the past decades (Rockett et al., 2018, 2022).

Despite these limitations, the study findings have important clinical and policy implications for preventing late-life suicide. First, health care professionals, especially primary care physicians who have frequent contact with older adults, should routinely assess suicide risks among those suffering from health problems and depressive symptoms. Second, older adults who are at risk of suicide due to health problems should be provided physical and behavioral health services and other resources targeted at increasing physical comfort, decreasing depression, and improving the overall quality of life. Affordable and easily accessible behavioral health treatment focusing on effective suicide prevention strategies is needed for those at risk of suicide. Integration of depression care managers into primary care for late-life suicide risk reduction has a strong evidence base (Ding & Kennedy, 2021). Tele-medicine and tele-­psychiatry are also effective strategies to increase access to physical and mental health care among older adults with disability and transportation problems (Gentry et al., 2019).

Third, older adults who are at risk of suicide due to PH or other risk factors should be prevented from accessing lethal suicide means including firearms and large quantities of potentially dangerous medications. Family members and health care professionals in both primary care and mental health settings need to identify those at risk and should work together with older adults to develop safety protocols and restrict access to these means. Although the Second Amendment curtails legislation broadly restricting firearm access in the U.S., policy-level interventions that limit firearm access among those at risk of suicide may have some effect (Allchin et al., 2019; Mann & Michel, 2016).

Fourth, the finding that PH was the most prevalent suicide precipitant even in the 55–64 age group underscores the importance of improving these young-old individuals’ well-being by improving access to both physical and mental health care. A previous study found that among decedents younger than age 65 with PH, the likelihood of suicide is elevated in states with limited health care access and weaker gun control laws (Phillips & Hempstead, 2022). Research has also shown the importance of cultivating and maintaining meaning in life in promoting positive psychological factors, potentially conferring resilience, and reducing suicidal ideation, especially among those with chronic illnesses (Heisel & Flett, 2016; Lutzman & Sommerfeld, 2023).

In conclusion, preventing suicide among older adults who are suffering from debilitating illnesses and associated depression requires a comprehensive approach that addresses both their physical and mental well-being. Effective suicide prevention strategies need to include enhanced health care support ensuring access to high-quality health care services and effective pain management; appropriate mental health care options including pharmacotherapy and/or psychotherapy; increased resources for quality long-term and palliative care; and development and updating, as needed, of safety and means limitation plans; and effective firearm regulations aimed at reducing suicide. Increased community-based support, through aging service and faith-based organizations, to meet the needs for instrumental/basic activities of daily living and to reduce social isolation, loneliness, and other life stressors is also needed.
